# Improvement of the rituximab–induced cell death by potentiation of the store-operated calcium entry in mantle cell lymphoma cell lines

**DOI:** 10.18632/oncotarget.27063

**Published:** 2019-07-09

**Authors:** Isabelle Doignon, Olivier Fayol, Olivier Dellis

**Affiliations:** ^1^Interactions Cellulaires et Physiopathologie Hépatique, INSERM UMR-S 1174, Paris, France; ^2^Université Paris-Sud, Université Paris Saclay, Paris, France

**Keywords:** mantle cell lymphoma, rituximab, store-operated calcium entry, potentiation, apoptosis

## Abstract

Mantle Cell Lymphoma (MCL) is one of the worst lymphomas with a median overall survival of 3 to 4 years. Even if the use of rituximab was a great step in therapy, patients commonly develop resistance and relapse. New therapies or complement of existing therapies should be developed. Using spectrofluorimetry, we found that the resting cytosolic Ca^2+^ ion concentration [Ca^2+^]_cyt_ of MCL patients cells and MCL cell lines was increased. This increase is correlated with a larger store-operated calcium entry (SOCE) amplitude which is responsible for the Ca^2+^ ions influx. Furthermore, using a SOCE potentiating agent, we demonstrated that in the MCL Rec-1 cell line, the SOCE is already activated in resting conditions. Interestingly, this potentiating agent alone, by disturbing the SOCE, induced the apoptosis of Rec-1 cells with the same efficacy than rituximab. The use of the potentiating agent in addition to rituximab strengthens the rituximab-induced apoptosis of rituximab-sensitive Granta-519 and Rec-1 cells. However, this potentiating agent cannot convert the Jeko-1 rituximab-resistant to a rituximab-sensitive cell line. Our results confirm that the use of compound acting on the Ca^2+^ homeostasis could be a new target of interest in complement to existing therapies.

## INTRODUCTION

Rituximab (RTX), a chimeric IgG1 anti-CD20 monoclonal antibody, allows the depletion of B cells by inducing their apoptosis by complex pathways [1–3]. It has been commonly used for several years to treat Non-Hodgkin Lymphomas (NHL) in complement to previously established chemotherapies like CHOP [4]. However, patients could develop resistance and relapse [5]. Furthermore, chemotherapies + RTX could be more toxic for patients [6]. Thus, new combinations of chemotherapies or new ways of therapies are under investigation.

Cross-linking of CD20 by RTX induces a massive Ca^2+^ ion influx, known as the Store-Operated Calcium Entry (“SOCE”) and is crucial for apoptosis [7]. In B lymphocytes, stimulation of the B cell receptor is the main way to activate the SOCE: it induces the synthesis of inositol 1,4,5-trisphosphate, allowing the release of Ca^2+^ ions trapped in the endoplasmic reticulum (ER). This, in turn, induces the oligomerization of ER-membrane STIM1 proteins, which directly open the plasma membrane Orai1 channels, allowing the massive Ca^2+^ influx known as the SOCE [8]. Noteworthy, the BCR signalling pathway is deregulated in some cancer like the Mantle Cell Lymphoma [4].

It is known that resistance to RTX could be due to a decrease of CD20 cell surface expression [9]. As consequences, the RTX-induced Ca^2+^ mobilization and apoptosis are reduced in RTX-resistant Raji cells [9]. Recently, it has been shown that the SOCE is implied in CD95-dependent RTX-induced apoptosis of follicular lymphoma and diffuse large B cell lymphoma cells [10]. Thus, it is clear that Ca^2+^ mobilization is crucial for RTX effects.

Since the discovery of the main proteins allowing the SOCE (Orai1 and STIM1), the characterization of SOCE modulators have emerged enough attracting by pharmaceutical companies like Hoffman-La Roche or GSK [11]. Indeed, the SOCE had also been shown to be implied in autoimmune disorders, inflammation and cancers [11]. Molecules acting on the SOCE could therefore be of interest to help/reinforce the RTX-induced effects.

In the past two decades, many SOCE inhibitors have been characterized [12–14]. Recently, we isolated a SOCE potentiating compound (Methyl DiEthyl Borinate, MDEB [15]). MDEB potentiates the SOCE of the T cell line Jurkat after TCR stimulation [15]. Surprisingly, this potentiation did not result in a larger activation of the cells, but to a significant induced apoptosis. Furthermore, MDEB is not toxic for non-stimulated cells. Thus, this compound allows elimination of activated cells [15].

As RTX is known to induce the Ca^2+^ mobilization in Mantle Cell Lymphoma (MCL) cells [4], we here studied the effect of MDEB-induced SOCE potentiation in complement to RTX. MCL is an aggressive B-cell lymphoma which represents around 6 % of the non-Hodgkin lymphomas (NHL, [16]) and has a bad prognosis with a median overall survival of 3-4 years [17]. MCL derives from naïve pre-germinal B cells that proliferate in the mantle zone of the germinal centres found in the lymph node. This proliferation is mainly due to the over-expression of cyclin D1, which is absent in B cells. Cyclin D1 plays an important role in the G2-S transition of the cell cycle. Other mutations are also present (p53 for example) [18]. As MCL cells express the pan-B cell antigen CD20, it allows the use of RTX for treatment [18, 19].

Here we reported that the Ca^2+^ homeostasis of MCL patient cells and some commonly used MCL cell lines is abnormal due to a SOCE amplitude increase. Furthermore, our strategy to high-jack the Ca^2+^ homeostasis by using a potentiating SOCE agent was able to improve the RTX-induced apoptosis and could represent a new complementary way to control this kind of lymphoma. However, we also observed that the SOCE potentiation of a RTX-resistant MCL cell line could not convert it to a sensitive one.

Thus, we confirm that the SOCE could represent a new target in the treatment of some cancer like lymphomas.

## RESULTS

### The Ca^2+^ homeostasis of MCL patients’ blasts is “abnormal”

It is well admitted that the resting [Ca^2+^]_cyt_ of cells is near 100 nM in physiological conditions [20]. Using spectrofluorimetry we investigated the Ca^2+^ homeostasis of cells from four MCL patients.

**Figure 1:**
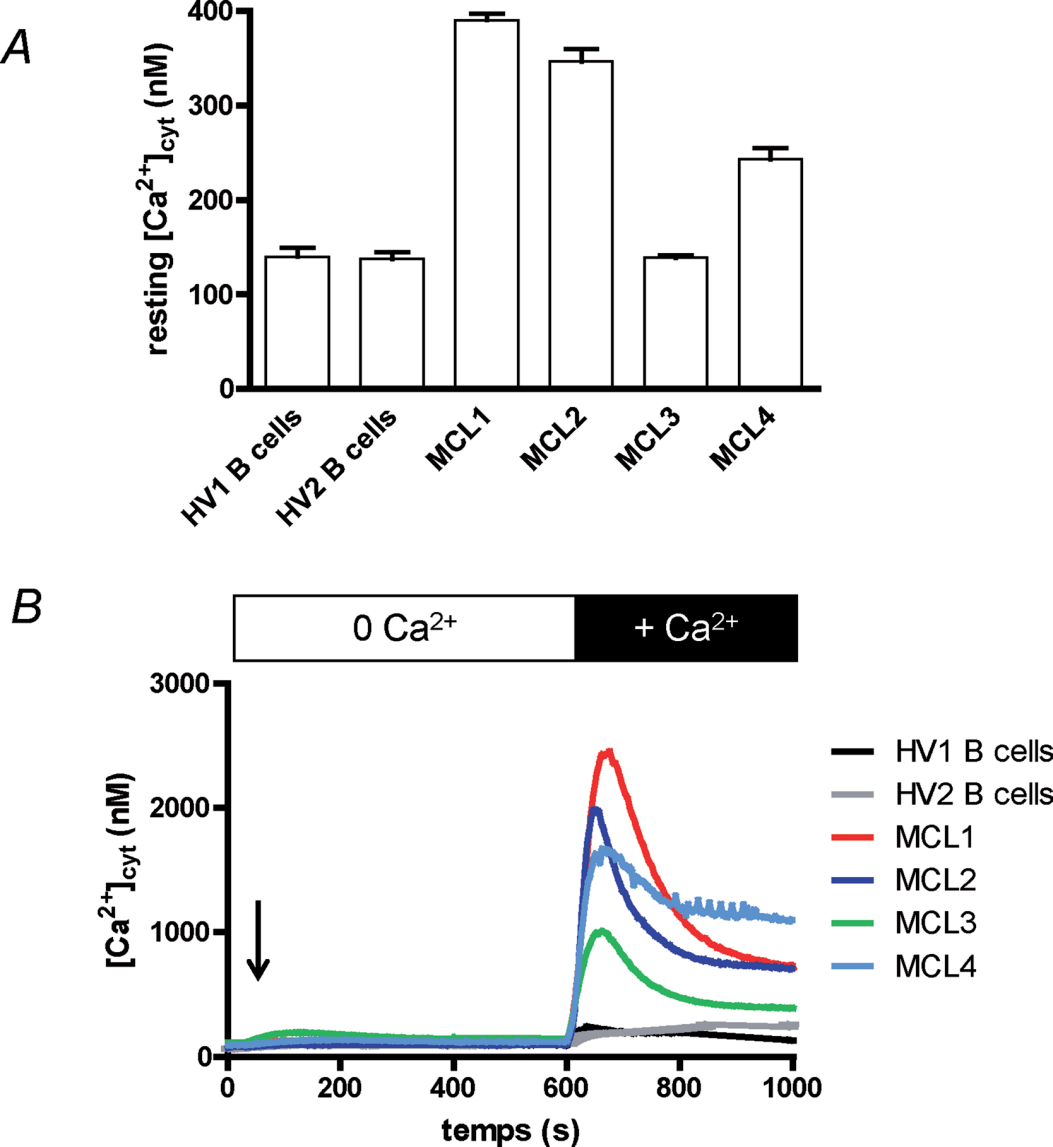
Ca^2+^ homeostasis of MCL lymphoblasts is different from circulating B cells’. **(A)** Healthy volunteer circulating B (“HV1” and “HV2”) cells and MCL lymphoblasts from four MCL patients (“MCL1” to “MCL4”) were placed in Hepes-Buffer Saline (HBS) medium supplemented with 1 mM extracellular CaCl_2_ and resting [Ca^2+^]_cyt_ was measured by using Indo-1 fluorescence. Recordings last 2 min, a mean resting [Ca^2+^]_cyt_ was calculated for each cell type and were done at least three times. **(B)** Cytosolic Ca^2+^ concentration ([Ca^2+^]_cyt_) variations were measured at first in Ca^2+^-free HBS medium. Cells were treated for 10 min with thapsigargin (1 μM, arrow) to allow Ca^2+^ release from ER and opening of the SOCE channels. After 10 min, 1 mM CaCl_2_ was added, allowing Ca^2+^ entry through the SOCE channels. [Ca^2+^]_cyt_ was measured by using Indo-1 fluorescence. Results are representative for 5 experiments.

The resting [Ca^2+^]_cyt_ of two healthy volunteers (HV) circulating B cells was ≈ 150 nM (140 ± 9 and 138 ± 7 nM for “HV1” and “HV2” respectively, n = 3 for both, Figure 1A). In contrast, the resting [Ca^2+^]_cyt_ of lymphoma cells from the four MCL patients was very heterogeneous: one was not statistically different from the value obtained with healthy donor B cells (139 ± 2 nM, n =3, “MCL3”), the three others were largely and significantly increased: 390 ± 7, 347 ± 13 and 243 ± 12 nM respectively for MCL patients MCL1, MCL2 and MCL4 (n = 3, p < 0.01),.

As the resting [Ca^2+^]_cyt_ of leukocytes is mainly driven by the Ca^2+^ influx, known as Store-Operated Ca^2+^ Entry (SOCE) in lymphocytes, we next studied the variation of [Ca^2+^]_cyt_ (Δ[Ca^2+^]_cyt_) under the stimulation by thapsigargin (TG). TG, by inhibiting the Ca^2+^ pumps responsible of Ca^2+^ ion accumulation in the endoplasmic reticulum (ER), induces the passive release of Ca^2+^ ions by the ER, and next the opening of the plasma membrane Orai1 channels resulting in a SOCE. No significant differences were observed between the four patient lymphoma cells and the healthy donor B cells (Figure 1B) in extracellular Ca^2+^-free conditions, meaning the ER Ca^2+^ release is not different. After ~ 10 min, the [Ca^2+^]_cyt_ returned to values prior TG addition, due to the Ca^2+^ ion efflux from the cells. Readdition of extracellular Ca^2+^ ions allowed their entry in the cell by the SOCE, resulting in an increase of [Ca^2+^]_cyt_. As clearly showed in Figure 1B, the Δ[Ca^2+^]_cyt_ was larger with the four MCL patient cells when compared to the response of circulating B cells from healthy volunteers. Interestingly, lymphoma cells with the higher resting [Ca^2+^]_cyt_ were also the cells with the largest SOCE (Patient MCL1). Thus, the MCL1 cell peak Δ[Ca^2+^]_cyt_ reached 2339 ± 34 nM *vs.* 144 ± 7 nM for HV1 cells (p < 0.01, n=3).

Furthermore, the [Ca^2+^]_cyt_ increase after Ca^2+^ ion readdition was totally blocked by 30 μM 2-APB (Supplementary Figure 1, example of MCL3 cells) a concentration known to block the SOCE induced by TG [14].

Despite a low number of MCL patients, it seems that the SOCE is increased in the blast cells compared to circulating B cells, and as a consequence the resting [Ca^2+^]_cyt_ could be also increased. As our patients were quickly treated by chemotherapy, we decided to continue our work on three commonly used MCL cell lines: Rec-1, Jeko-1 and Granta-519. First, we wanted to verify if the same Ca^2+^ homeostasis disturbances exist in these cell lines.

### Rec-1 and Granta518 cells have an elevated resting [Ca^2+^]_cyt_

**Figure 2:**
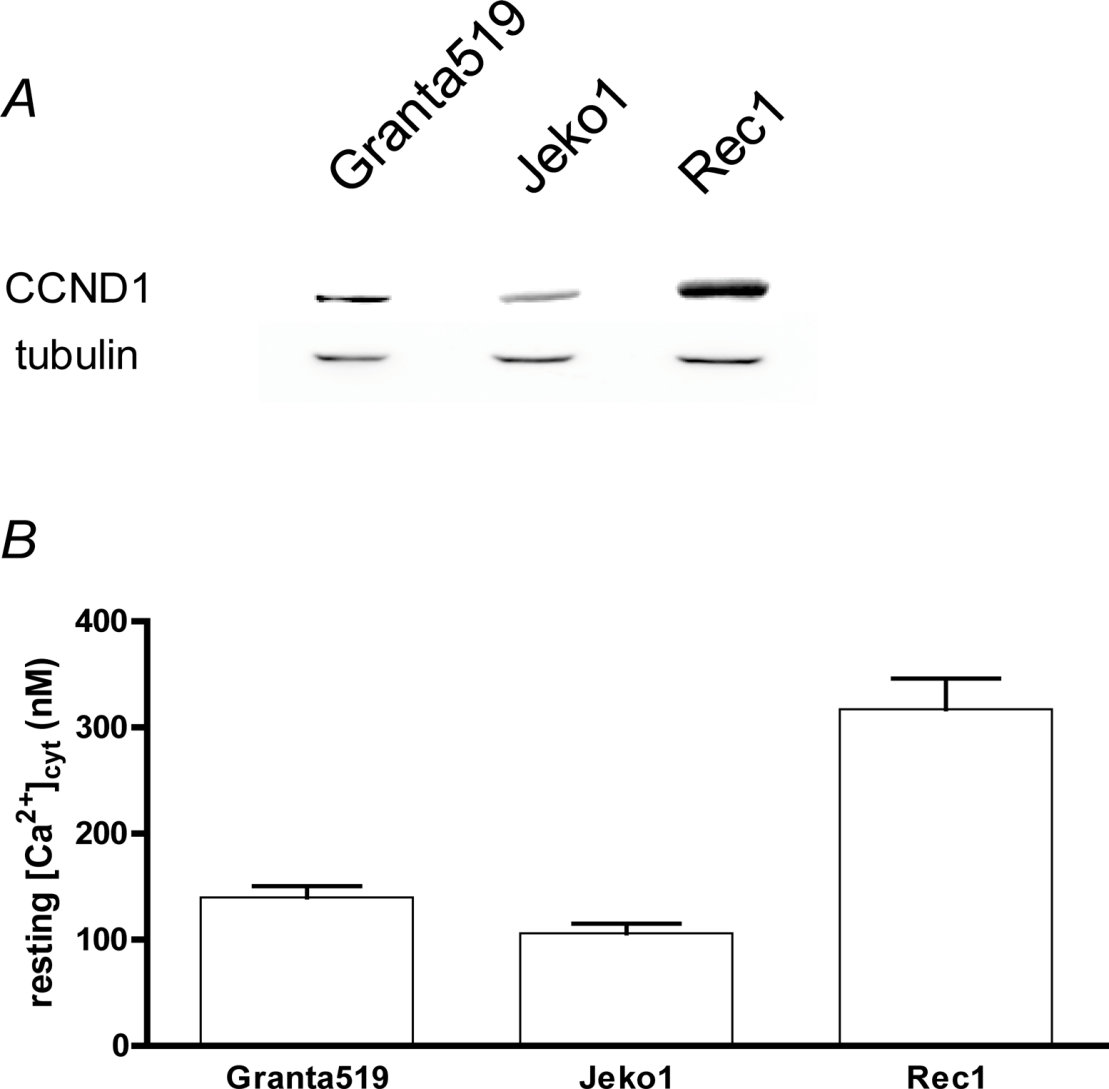
MCL cell lines express Cyclin D1 and can show an increased resting [Ca^2+^]_cyt_. **(A)** Western-blotting of cyclin D1 (CCND1) and tubulin expressions in three commonly used MCL (Granta 519, Jeko-1 and Rec1) cell lines. **(B)** Resting [Ca^2+^]_cyt_ of the three MCL cell lines was heterogeneous. Resting [Ca^2+^]_cyt_ was measured as in Figure 1A.

MCL is characterized by the expression of cyclin D1 by MCL blasts [21]. To verify the cyclin D1 expression, a western-blot was performed on the three MCL cell lines (Granta-519, Jeko-1 and Rec-1). The three MCL cell lines expressed different amounts of cyclin D1, with Rec-1 cells expressing the most (~5.5 and ~3.5 - fold more than Jeko-1 and Granta-519 cells respectively, Figure 2A).

As shown in Figure 2B, the resting [Ca^2+^]_cyt_ of the three MCL cell lines was heterogeneous: 106 ± 9 nM, 140 ± 11 nM and 278 ± 20 nM (n = 10) respectively for Jeko-1, Granta-519 and Rec-1 cells. Thus the resting [Ca^2+^]_cyt_ of Granta-519 and Rec-1 cells is significantly higher than the one of Jeko-1 cells (p<0.05 and <0.01 respectively).

Taken together, these results clearly showed that the Ca^2+^ homeostasis of MCL cell lines is not homogenous, and that the Rec-1 cell line presents a resting [Ca^2+^]_cyt_ similar to what is observed with activated lymphocytes [15]. Interestingly, the MCL cell lines were as variable as MCL blasts from patients.

### Rec-1 and Granta-519 cells have an elevated SOCE

**Figure 3:**
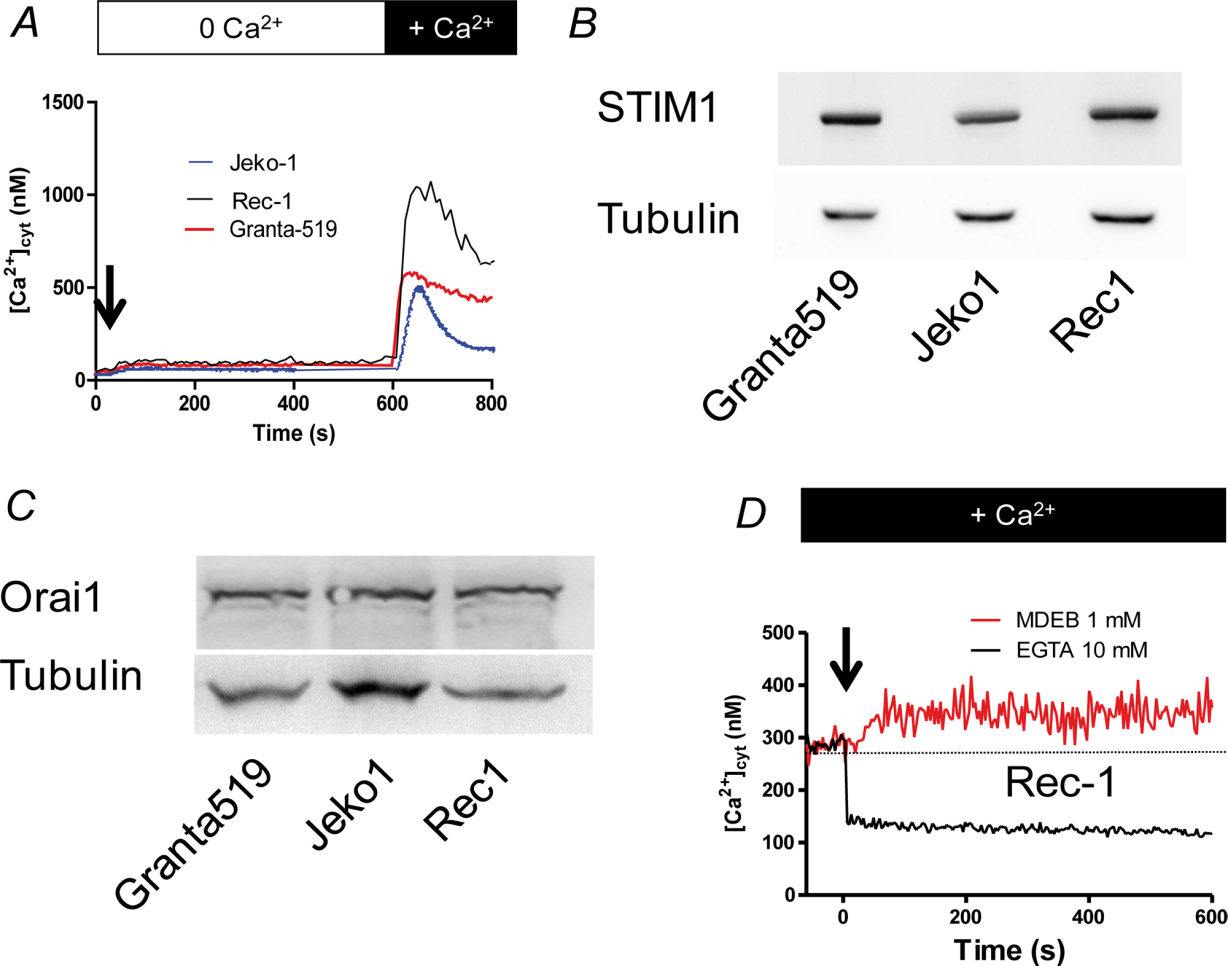
The SOCE is large in MCL cell lines. **(A)** MCL cells were placed in a Ca^2+^-free HBS medium and stimulated by 1 μM TG during 10 min to induce the Ca^2+^ release by the ER and the opening of SOC channels. Then 1 mM CaCl_2_ was added to visualize the SOCE. [Ca^2+^]_cyt_ was measured as in figure 1B. **(B)** Western-blotting of STIM1 expression in the three MCL cell lines, showing no significant difference among MCL cell lines. **(C)** Western-blotting of Orai1 expression in the three MCL cell lines, showing no significant difference among MCL cell lines. **(D)** Addition of the SOCE-potentiating agent MDEB (1 mM, arrow) increases the resting [Ca^2+^]_cyt_ of Rec1 cells. On the contrary, addition of 10 mM EGTA to chelate extracellular Ca^2+^ ions induced a fast decrease. Resting [Ca^2+^] was measured as in figure 1A. Dotted line represents the untreated cell [Ca^2+^]_cyt_.

**Figure 4:**
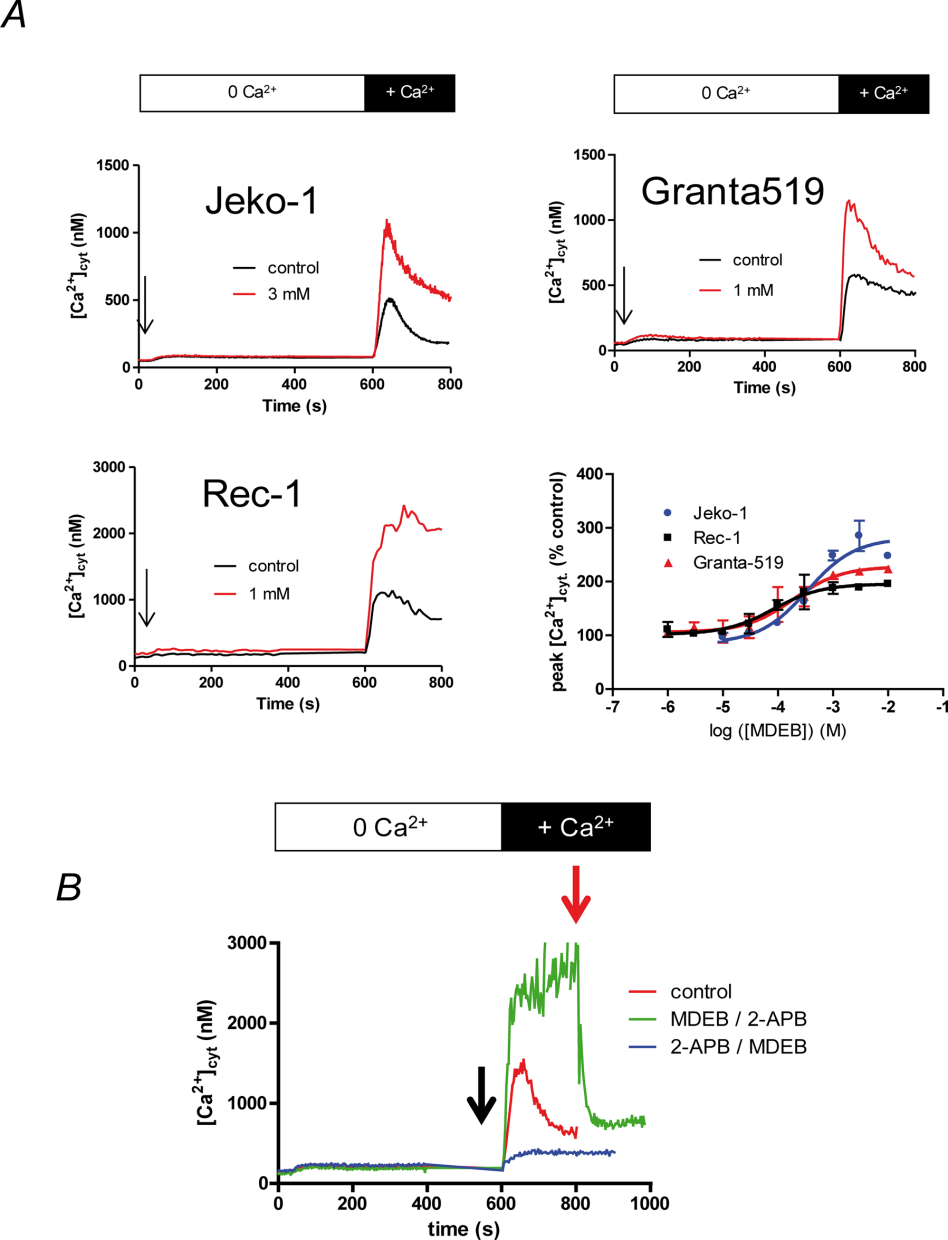
MDEB potentiates the SOCE of the MCL cell lines with different affinity. **(A)** The three cell lines were placed in Ca^2+^-free HBS medium and stimulated 10 min with TG 1 μM. 1 mM CaCl_2_ was then added to induce the SOCE. Different concentration of MDEB were added 30 s prior CaCl_2_. Only the MDEB concentrations with maximal potentiating effect were shown in this figure. Traces are representative of more than 3 experiments. The dose-response curve was obtained by expressing the peak of [Ca^2+^]_cyt_ increase under MDEB treatment as percentage of [Ca^2+^]_cyt_ increase obtained in absence of MDEB. MDEB dose-response curves could be fitted with a simple sigmoidal curve, giving potentiation constant of 338 ± 38 nM (Jeko1), 96 ± 19 nM (Granta-519) and 91 ± 24 nM (Rec-1 cells, n = 3 for each). **(B)** Competition of 1 mM MDEB and 30 μM 2-APB on Rec-1 cell SOCE. The same protocol was used as in figure 4A except that 2-APB or MDEB was added 30 s prior CaCl_2_ (black arrow), then MDEB or 2-APB was added at 200s after CaCl_2_ (t = 800 s, red arrow).

As with the resting [Ca^2+^]_cyt_, the three MCL cell lines responded differently to TG after Ca^2+^ readdition, especially Rec-1 cells (Figure 3A). Thus, the peak of Δ[Ca^2+^]_cyt_ was higher in Rec-1 cells than in Jeko-1 and Granta-519 cells: 863 ± 18 nM (n = 3, p < 0.01) *vs.* 423 ± 16 nM (n = 3, p < 0.01) and 484 ± 14 nM (n=3, p < 0.01) respectively. The Δ[Ca^2+^]_cyt_ rise after Ca^2+^ readdition was inhibited by 30 μM 2-APB (a classical inhibitor of SOCE at this concentration [22]) added 30 s prior Ca^2+^ ions in the three cell lines (Figure 4B for Rec-1 cells). This result confirms that the Δ[Ca^2+^]_cyt_ rise was due to a SOCE induced by TG and that Rec-1 cells possess an increased SOCE when compared to other MCL cell lines. Noteworthy, even if the [Ca^2+^]_cyt_ rise was similar between Jeko-1 and Granta-519 cells, the kinetics of the decay was different: fast for Jeko-1 cells SOCE, very faint for Grant-519’s, meaning that the Ca^2+^ rise in Jeko-1 cells is largely transient, but sustained in Granta-519 cells (and in Rec-1 cells because the [Ca^2+^]_cyt_ stayed at a high level after a partial decay).

As the two main proteins responsible for the SOCE in B cells are STIM1 and Orai1, we next performed western-blotting to study their expression in the MCL cell lines (Figure 3B and 3C). Clearly, the 3 MCL cell lines express similar amounts of Orai1 and STIM1 proteins. Thus, the differences of Ca^2+^ homeostasis (at least resting [Ca^2+^]_cyt_ and SOCE) we observed between the MCL cell lines are not directly linked to a difference of Ca^2+^ transporter expression.

Rec-1 cell line is the cell line with the largest values of resting [Ca^2+^]_cyt_ and SOCE amplitude without any significant difference in Orai1 and STIM1 expression compared to the other MCL cell lines. Thus, we wondered if the Ca^2+^ homeostasis changes could be due to an already activated SOCE in resting conditions allowing a passive extracellular Ca^2+^ influx. To assess if the SOCE is already opened, we next added 1 mM MDEB, a compound we recently characterized with only potentiating capacity on activated-SOCE to Rec-1 cells in presence of 1 mM extracellular CaCl_2_ [15]. As clearly showed in Figure 3D, MDEB induced a ~60 nM increase of resting [Ca^2+^]_cyt_ (351 ± 39 nM after 10 min of MDEB *vs.* 287 ± 17 nM before MDEB addition, n = 3 for both). On the opposite, when 10 mM EGTA was added to chelate the extracellular Ca^2+^ ions, the resting [Ca^2+^]_cyt_ decreased instantaneously. As MDEB acts only on activated-SOCE [15], and that extracellular EGTA drastically reduced [Ca^2+^]_cyt_, our results showed that in resting conditions, Rec-1 cell SOCE is already activated and Rec-1 cells possess a “leaky SOCE”.

### MDEB potentiates the MCL cell SOCE

We next tested and verified the effect of MDEB on the SOCE on the three MCL cell lines at different concentrations (Figure 4). The SOCE was potentiated by progressively increasing MDEB concentrations with a maximal effect above 1 (Granta-519 and Rec-1 cells) or 3 mM (Jeko-1 cells, Figure 4A and 4B). Furthermore, the fit of the dose-response curves allowed the calculation of an apparent potentiation constant *K* of 338 ± 38, 96 ± 19 and 91 ± 24 nM for Jeko-1, Granta-519 and Rec-1 cells respectively (n = 3 for each, Figure 4B).

MDEB capacity to potentiate the SOCE was also different between the cell lines (Figure 4A). Thus, at the optimal MDEB concentration, the SOCE was potentiated by 266 ± 16, 217 ± 4 and 191 ± 4 % for Jeko-1, Granta-519 and Rec-1 cells respectively (n = 3 for each).

To confirm that MDEB targeted the SOCE, we also performed competition experiments with 2-APB as we previously done on Jurkat T cells [15]. As clearly showed in Figure 4B on Rec-1 cells (same for the two other MCL cell lines, not shown), 30 μM 2-APB inhibited the Ca^2+^ rise after Ca^2+^ readdition (blue curve). MDEB (added at t = 800 s) was not able to counteract the 2-APB inhibition anymore and had no potentiating effect. On the contrary, when 1 mM MDEB was added 30s prior extracellular Ca^2+^ ion readdition, the Ca^2+^ rise was potentiated (green curve). However, since 30 μM 2-APB was added, the [Ca^2+^]_cyt_ quickly fell.

Noteworthy, due to an already increased SOCE, Rec-1 cells were the only cells where MDEB was able to maintain a ~ 2 μM [Ca^2+^]_cyt_ (Figure 4A), in Granta-519 and Jeko-1 cells, after reaching a peak [Ca^2+^]_cyt_, there was a decay.

### RTX stimulation increases [Ca^2+^]_cyt_

**Figure 5:**
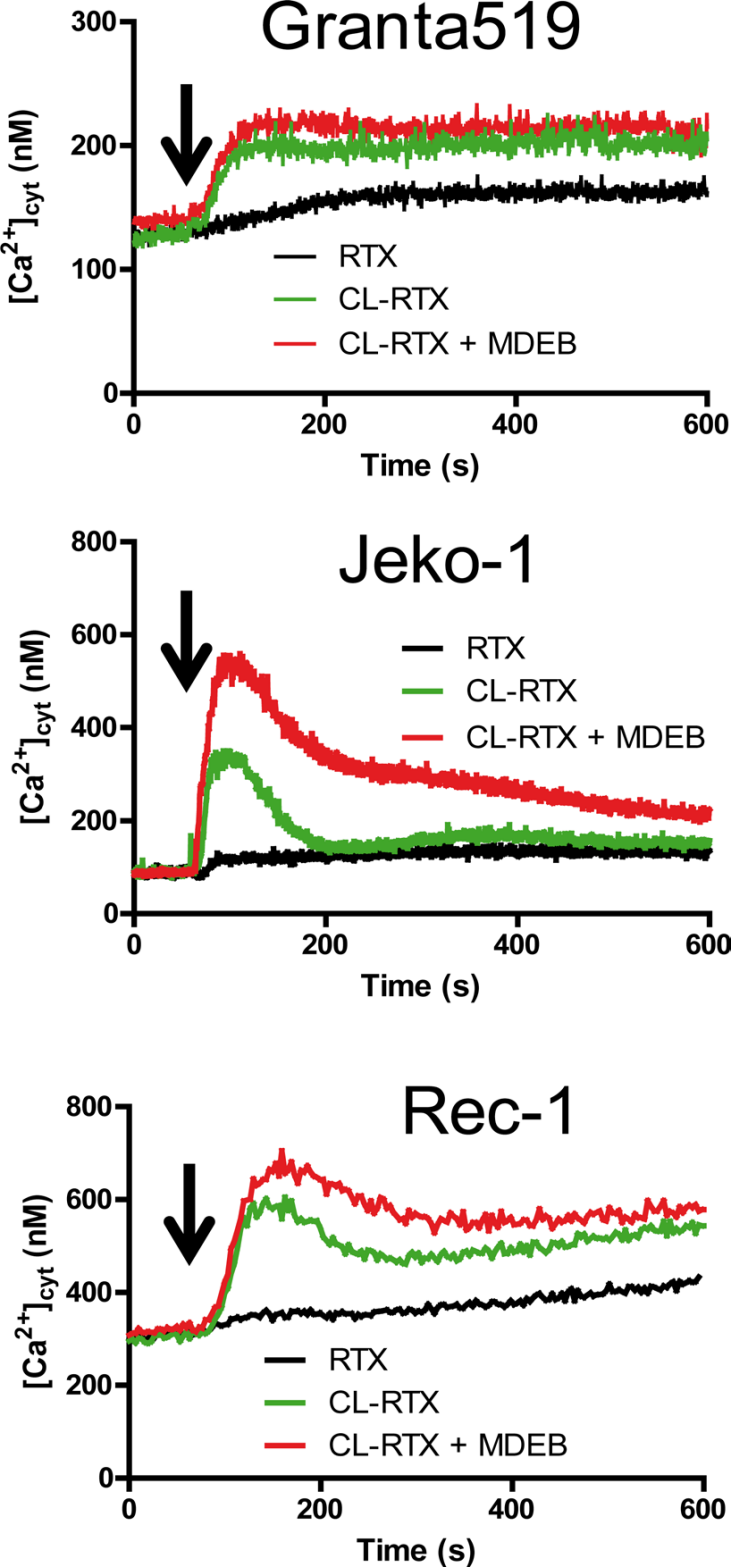
RTX and CL-RTX treatment differently modify the Ca^2+^ homeostasis of MCL cell lines. All cell lines were placed in 1 mM CaCl_2_-containing HBS. RTX (1 μg/ml), F(ab')_2_ fragment cross-linked RTX (CL-RTX, 1 μg/ml RTX + 10 μg/ml F(ab’)_2_) or CL-RTX + MDEB (1 mM MDEB) were added as indicated by an arrow. [Ca^2+^]_cyt_ was measured as in Figure 1B.

Next the three MCL cell lines were stimulated in presence of extracellular 1 mM CaCl_2_ with different combination of 10 μg/ml RTX: alone or previously cross-linked with a F(abʼ)_2_ fragment (in presence or absence of 1 mM MDEB (Figure 5)).

RTX alone was only able to slowly and weakly mobilize cytosolic Ca^2+^ (black curves) in the three cell lines. As shown previously on several Burkitt Lymphoma cell lines [23], F(abʼ)_2_ fragment cross-linked RTX (1x10 ratio, “CL-RTX”) was more effective than RTX alone to induce the cell response: thus the CL-RTX-induced Ca^2+^ mobilization (green traces) could be transient (Jeko-1 cells) or maintained during several minutes (Granta-519 cells). The profile observed with Rec-1 cells was quietly a mix of the two possibilities: after a rapid ~300 nM increase, there was a decay (~ -100 nM) followed by a constant [Ca^2+^]_cyt_ rise.

MDEB addition in the same time as CL-RTX increased the Δ[Ca^2+^]_cyt_ in the different cell lines (red traces), especially in Jeko-1 cell lines which had a transient CL-RTX response Thus, in Jeko-1 cells, the CL-RTX-induced peak [Ca^2+^]_cyt_ reached 510 ± 15 nM in presence of 3 mM MDEB (*vs.* 327 ± 14 nM in its absence, n = 3 for both). After 10 min of stimulation, the [Ca^2+^]_cyt_ of Jeko-1 cells remained higher in presence of MDEB (216 ± 10 *vs.* 155 ± 4 nM, n = 3 for both) but continuously decreased.

In Granta-519 cells, 1 mM MDEB had no statistical effects during the 10 min of the experiment even if a faint increase is viewable: [Ca^2+^]_cyt_ reached 214 ± 16 *vs.* 200 ± 4 nM in presence or absence of MDEB respectively (n = 3 for both).

In Rec-1 cells, the CL-RTX – induced peak [Ca^2+^]_cyt_ was significantly increased by 1 mM MDEB: 669 ± 34 *vs.* 582 ± 17 nM (n = 3 for both, n < 0.01). After 10 min of treatment, even if the MDEB-induced increase was weaker, it was still significant: 582 ± 15 vs. 541 ± 10 nM (n=3 for both). Interestingly, MDEB presence did not impair the constant increase of [Ca^2+^]_cyt_ induced by CL-RTX.

From these results, we showed that RTX alone could not markedly mobilize the Ca^2+^ ions. However, when cross-linked with F(ab')_2_ fragment, RTX could mobilize Ca^2+^ ions to different levels according to the cell line, with transient (Jeko-1 cell line) or sustained effect (Granta-519 and Rec-1 cell lines). Furthermore, MDEB was significantly able to potentiate the CL-RTX Ca^2+^ increase in two of MCL cell lines.

### MDEB increases the RTX-induced apoptosis of Granta-519 and Rec-1 cells

**Figure 6:**
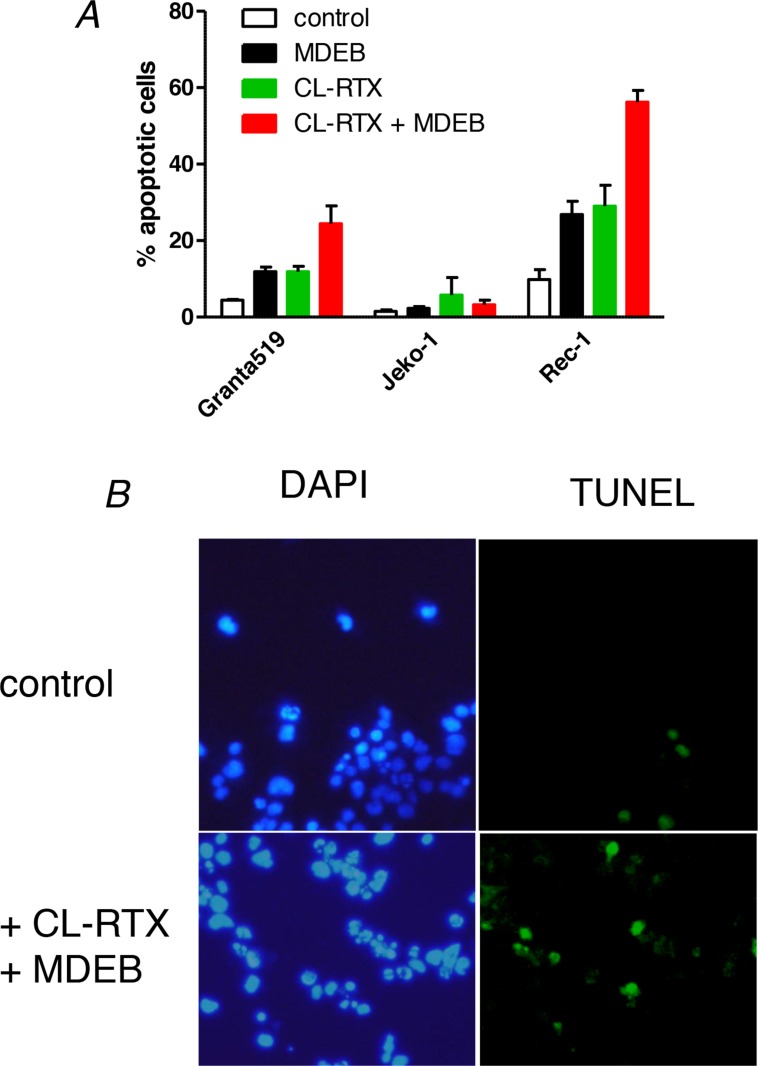
MDEB reinforce RTX-induced apoptosis of RTX-sensitive cell lines. **(A)** The three cell lines were stimulated during 24h with MDEB (1 mM), CL-RTX (1 μg/ml RTX + 10 μg/ml F(ab’)_2_) or not stimulated. After 24h, cells were centrifuged and stained with DAPI to visualize the nuclei and with the TUNEL method to visualize apoptotic cells. More than 200 cells were counted on different fields and the percentage of apoptotic cells was calculated as number of TUNEL-positive cells on DAPI-positive cells. **(B)** Representative pictures of Rec-1 cells in control conditions or after 24h of treatment with MDEB + CL-RTX. DAPI staining is on the left, TUNEL’s on the right. Scale bar = 10μm.

In a previous work we showed that MDEB was able to induce the apoptosis of cells unable to regulate their resting [Ca^2+^]_cyt_ after a stimulation [15]. As it is well established that CL-RTX induces more apoptosis than RTX alone [24], we next stimulated the cell lines during 24h with CL-RTX alone, CL-RTX + MDEB or MDEB alone and performed a TUNEL analysis (Figure 6)

The Jeko-1 cell line was clearly resistant to the apoptosis induced by CL-RTX or CL-RTX + MDEB. Indeed, the percentage of apoptotic cells never overtook 6% in the different conditions. (Figure 6A)

On the contrary, the two other MCL cell lines were sensitive to CL-RTX and MDEB potentiated the CL-RTX-induced apoptosis, with the Rec-1 cells showing the largest effects (Figure 6A). Thus, MDEB and CL-RTX significantly increased the apoptosis of Granta-519 cells: 12 ± 1 (p < 0.05) and 12 ± 1 % (p < 0.05) respectively *vs.* 4 ± 0% in untreated cells. Under CL-RTX + MDEB treatment, the number of apoptotic cells reached a percentage of 25 ± 3 % (p < 0.01). Thus, the effects of MDEB and CL-RTX were additive and not synergistic (12 + 12 ≈ 25%).

Rec-1 cell line showed a significant number of apoptotic cells under resting culture conditions when compared to the two other cell lines: 10 ± 3% vs. < 4 % in mean (typical picture in Figure 6B). Addition of MDEB or CL-RTX significantly increased by ~3 fold the percentage of apoptotic cells: 27 ± 3 (p < 0.05) and 29 ± 5% (p < 0.05) respectively. Under MDEB + CL-RTX treatment, this number raised to 56 ± 3 % (p < 0.01, typical picture in Figure 6B). As for Granta-519 cells, the effects of MDEB and CL-RTX were only additive.

## DISCUSSION

During the last decade, anti-CD20 receptor antibodies in complement to “classical” cytotoxic drugs have been increasingly used in treatment of B-NHL with a remarkable improvement of survival. However, the use of RTX, the main anti-CD20 antibody, leads to appearance of resistance and relapse [5]. MCL is one of the deadliest diseases among human B-NHL and even if the use of RTX had been a major progress, it is still not sufficient.

Agents active on Ca^2+^ transporters probably deserve further investigations based on a new analysis of Ca^2+^ homeostasis disturbances. Common feeling is that agents active on Ca^2+^ homeostasis will not have enough selectivity. This is not true if we explore Ca^2+^ alterations specific of a disease. Ten years ago, the two main proteins responsible for the SOCE of B and T lymphocytes, Orai1 and STIM1 were characterized [25, 26]. Thus the opening of Orai1 channels after the Ca^2+^ release by the ER allows a massive Ca^2+^ influx known as SOCE that activates calmodulin and thereafter NFAT to induce the cell proliferation [20]. Beside this role in signaling, the SOCE also allows the replenishing of the ER with Ca^2+^ ions, which are needed for the ER homeostasis. The absence of a functional Orai1 essentially induces a severe combinated immunodeficiency, without affecting other major organ functions [27]. Thus, targeting Orai1 is of interest to control the activation and the proliferation of cells of the lymphocyte lineage. Thereafter, two approaches could be considered according to the presence of Ca^2+^ disturbances, inhibition or potentialisation of the SOCE.

Our work clearly showed that stimulation of Mantle Cell Lymphoma cell lines by RTX induces the ER Ca^2+^ release and the SOCE. These results confirm previous results obtained on cell lines from other lymphomas of B cell origin [10, 28]. Inhibition of the SOCE by Orai1 down-expression or classical inhibitors enhance the RTX-induced apoptosis on Follicular Lymphoma and Diffuse Large B Cell lymphoma cell lines [10]. In this configuration, and on these cells, the inhibition of the SOCE can induce an ER stress, known to lead to apoptosis [29]. Indeed, Ca^2+^ ions are necessary to multiple functions of the ER like the protein folding and processing [30]. ER Ca^2+^ ion depletion by TG is known to trigger ER stress leading to cell death [30, 31]. Nevertheless, the use of SOCE inhibitors targeting Orai1 channels should be used with care: indeed, even if Orai1 proteins are vital for lymphocyte activation and proliferation, these proteins are widely expressed in the body, where they probably play a role only in the replenishing of the ER.

In this work we showed that the Ca^2+^ homeostasis of MCL patient cells could be largely modified with an increase of the resting [Ca^2+^]_cyt_ and a larger SOCE amplitude. By comparison, the Ca^2+^ homeostasis of typical MCL cell lines is weakly modified, with a large heterogeneity. However, the Rec-1 cells, which is the closest cell line in term of Ca^2+^ homeostasis, possesses an already activated SOCE that can be amplified by the potentializing agent MDEB. Therefore, MDEB, alone, significantly induced Rec-1 cell apoptosis. Furthermore, in complement to RTX treatment, SOCE potentiation largely increased the Rec-1 cell apoptosis by maintaining an elevated [Ca^2+^]_cyt_. The same kind of uncontrolled and sustained increase leading to a Ca^2+^ overload could be obtained using Ca^2+^ ionophore ionomycin, which is known to induce the cleavage of caspase-3 in Jurkat cells and their apoptosis [32]. It is interesting to note that SOCE potentiation was not efficient on the RTX-resistant Jeko-1 cell line, probably because the variations of [Ca^2+^]_cyt_ induced by RTX are transient and not sustained. Therefore, even if the use of SOCE potentiating agent could represent an interest to induce the apoptosis of cells directly or after stimulation, it needs an activated SOCE. Thus SOCE potentializing agents could have the advantage over SOCE inhibitors to target only cells with an activated SOCE (due to Orai1 as shown in [15]) and not all the cells expressing Orai1 proteins.

This study confirms the interest of compounds acting on Ca^2+^ channels of cancerous cells to high-jack the Ca^2+^ homeostasis. They do not represent a new therapy but could be used in complement to existing therapies to enhance disturbances of Ca^2+^ homeostasis, leading to cell death. However, despite interesting properties, our potentiating agent needs millimolar concentrations to be efficient. We assume that the future synthesis of analogues acting at lower concentrations will greatly increase the interest for Ca^2+^ modulators in cancer treatment.

## MATERIALS AND METHODS

### Cell lines and primary blasts

Clinical samples were obtained from patients with an active leukemic form MCL who granted informed consent in accordance with ethic committee (Department of Hemato-oncology, Hôpital Saint Louis, Paris, France). The diagnostic of MCL was ascertained on lymph nodes biopsy based on the recommendation of the World Health Organization classification [33]. Peripheral Blood Monocyte Cells from MCL patients and healthy volunteers were separated by Ficoll-Hipaque density centrifugation. B cells of healthy volunteers and MCL patients were isolated using a B cell negative isolation kit to avoid B cell activation (Human B Cell Isolation Kit, MACS Miltenyi Biotec SAS, Paris France). The MCL patients showed a clear blood involvement by the lymphoma cells. BL-2 cells (Burkitt B lymphoma) were provided by Dr Pierre Busson, Rec-1, Jeko-1 and Granta-519 cells (mantle cell lymphoma) by Dr Vincent Ribrag (both at Institut Gustave Roussy, Villejuif, France). BL-2, Rec-1 and Granta-519 cell lines were basically maintained in RPMI-1640 medium (Lonza, Verviers, Belgium) supplemented with 10 % heat-inactivated fetal calf serum and 2 mM L-glutamine. Jeko-1 cells were maintained in the same medium, except that 20% heat-inactivated fetal calf serum was added.

### Cytosolic Ca^2+^ concentration

[Ca^2+^]_cyt_ was recorded by a fluorimetric ratio technique as in [14, 34]. Leukocytes were spun and resuspended at a density of 10^6^ cells/ml in Phosphate-Buffered Saline (PBS) supplemented with 1 mg/ml bovine serum albumin and incubated in the dark with 4 μM Indo-1-AM for one hour at room temperature under slow agitation. Cells were then centrifuged and resuspended in Ca^2+^-free Hepes Buffered Saline solution (HBS; 135 mM NaCl, 5.9 mM KCl, 1.2 mM MgCl_2_, 11.6 mM Hepes, 11.5 mM glucose adjusted to pH 7.3 with NaOH) prior to measurement. After centrifugation, 0.5 to 1 x 10^6^ cells were suspended in 2 ml HBS in a quartz cuvette and inserted into a spectrofluorophotometer (RF-1501 Shimadzu Corporation, Kyoto, Japan) connected to a PC computer (Dell Computer Corp., Montpellier, France). A temperature of 37°C was maintained by circulating water from a bath. Ultraviolet light of 360 nm was used for excitation of Indo-1, and emissions at 405 and 480 nm were recorded. Background and autofluorescence of the cell suspension were subtracted from the recordings. The maximum Indo-1 fluorescence (*R*_max_) was obtained by adding 1 μM ionomycin to the cell suspension in the presence of 10 mM CaCl_2_. Minimum fluorescence was determined following depletion of external Ca^2+^ with 5 mM EGTA. [Ca^2+^]_cyt_ was calculated according to the equation [Ca^2+^]_cyt_ = *K*_d_ (R-*R*_min_)/(*R*_max_-R), where *K*_d_ is the apparent dissociation constant of Indo-1-calcium complex (230 nM), and R is the ratio of fluorescence values recorded at 380 nm in absence and presence of 10 mM CaCl_2_ [34].

In many experiments, cells placed into a quartz cuvette, were treated with 1 μM thapsigargin (TG) during 10 min in Ca^2+^-free HBS to induce Ca^2+^ release from the ER and the opening of SOCE channels [14, 35]. Then, 1 mM CaCl_2_ was added to measure the change in [Ca^2+^]_cyt_ subsequently to Ca^2+^ influx [35]. In the dose-response experiments, different concentrations of MDEB were added 30s prior Ca^2+^ readdition [14, 15].

For resting cytosolic calcium concentration measurements (rest[Ca^2+^]_cyt_), cells were resuspended in 1 mM CaCl_2_ – containing HBS. Then recordings of Indo-1 fluorescence were done in the same conditions, still with Ca^2+^-containing HBS.

### Apoptosis detection by TUNEL method

We measured the apoptosis levels in the different cell lines with the “in situ cell death detection” kit (Roche Applied Science, Meylan, France [15]). Briefly, 100 000 cells/well were seeded in a 96-well plate and treated 24 h with RTX (10 μg/ml + 100 μg/ml F(ab')_2_) and MDEB 1 mM or left untreated. Then according to the manufacturer’s instructions, cells were spun, fixed, permeabilized and the Terminal transferase dUTP Nick End Labelling (TUNEL) reaction was performed during 60 min at 37°C in darkness. For visualization of results, an inverted epifluorescence microscope (Axioskop, Karl Zeiss, Le Pecq, France) was used at an excitation wavelength of 488 nm, and an emission wavelength of 545 nm. Cells were also loaded with 4ʼ, 6ʼ –diamidino-2-phenylindole (DAPI) to visualize the nuclei. Total cell number and TUNEL-positive cells were counted in several fields and the ratio of apoptotic cells was calculated. This experiment was repeated three times.

### Western-blotting

For cellular protein extraction, one million cells were lysed using RIPA buffer (50 mM Tris-HCl pH8, 150 mM NaCl, 1% Triton X100, 0.5% deoxycholate, 0.2% SDS and 0.2 mM EDTA) supplemented with an antiprotease pellet (cOmplete^TM^ Mini Protease Inhibitor Cocktail Tablets, Roche Diagnostics, Meylan, France). SDS-PAGE electrophoresis (10%) was subsequently performed (50 μg proteins/lane). Proteins from the gels were electrotransferred onto Hybond ECL membrane (Amersham-GE Healthcare, Velizy-Villacoublay, France). After blocking with 5% non-fat dry milk dissolved in Tris-buffered saline containing 0.1% Tween 20 (“Tris buffer”) for 1h at room temperature, blots were washed three times with Tris buffer and probed 3h with specific primary antibody in 5 % non-fat dry milk in Tris buffer. The primary antibodies used against Orai1 (1:1000), STIM1 (1:1000), cyclin D1 (1:500) and tubulin (1:3000) were from Sigma-Aldrich, Saint Quentin Fallavier, France). The anti-tubulin antibody was used to verify that equal amounts were loaded in each lane. Detection was performed using the enhanced chemiluminescence reagent (ECL Western blotting detection reagents, Amersham-GE Healthcare). Quantification of the bands was performed with the ImageJ software.

### Chemicals

Methyl-diethylborinate (MDEB), thapsigargin (TG) and ionomycin were from Sigma-Aldrich (Saint Quentin Fallavier, France). Rituximab (MabThera, Roche Diagnostics, Mannheim, Germany; stock solution 10 mg/ml) was a kindly gift of the Central Pharmacy of Hôpital Saint Louis (Paris, France). A goat anti-human F(abʼ)_2_ fragment was used for RTX crosslinking (Thermo Scientific, Villebon, France).

### Statistical analysis

Given values are representative of at least 3 independent experiments and are given as mean ± SEM. When used, a t-test < 0.05 is considered as significant.

## SUPPLEMENTARY MATERIALS AND FIGURES



## Abbreviations

MCL: Mantle Cell Lymphoma
[Ca^2+^]_cyt_: cytosolic calcium concentration
Δ[Ca^2+^]_cyt_: variation of [Ca^2+^]_cyt_
SOCE: Store-Operated Calcium Entry
RTX: rituximab
CL-RTX: cross-linked RTX
ER: endoplasmic reticulum
TG: thapsigargin
MDEB: methyl diethylborinate
HBS: Hepes-buffered saline
TUNEL: Terminal transferase dUTP nick end labelling
DAPI: 4’-6’-diamidino-2-phenylindole.
