# Supplementary material for: Improvement of the rituximab–induced cell death by potentiation of the store-operated calcium entry in mantle cell lymphoma cell lines
Source: Oncotarget. 2019 Jul 9;10(43):4466–78. doi: 10.18632/oncotarget.27063 (PMC6633894; doi:10.18632/oncotarget.27063)
Supplement: Supplementary file 1 [file oncotarget-10-4466-s001.pdf]

Improvement of the rituximab–induced cell death by potentiation of the store-operated calcium entry in mantle cell lymphoma cell lines

SUPPLEMENTARY MATERIALS

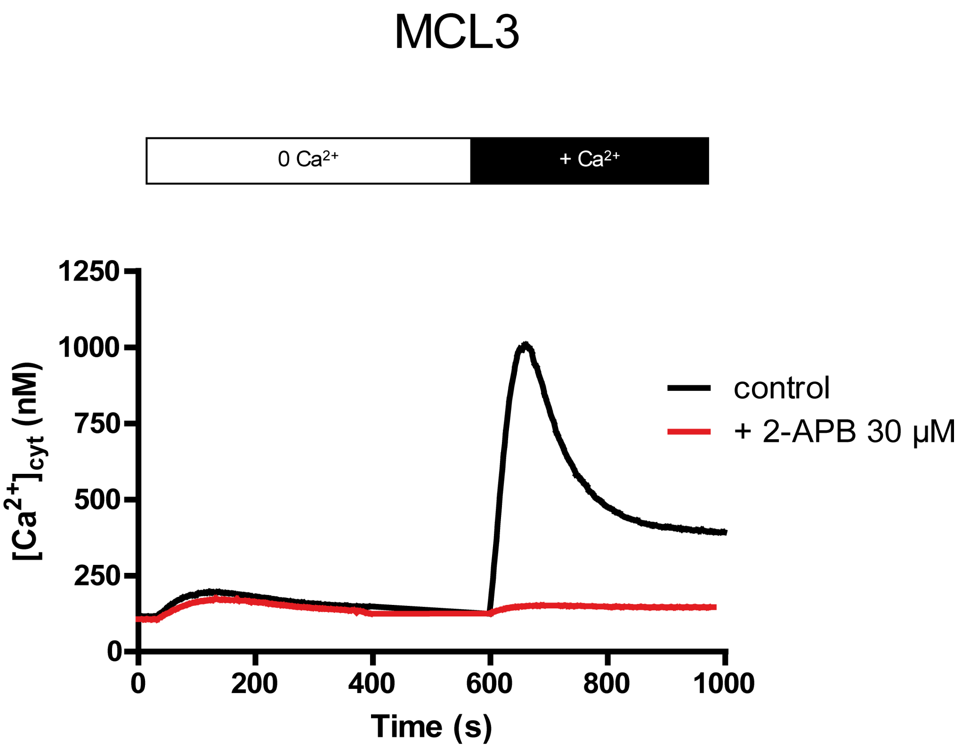

Supplementary Figure 1: 2-APB inhibit the SOCE of MCL3 patient blasts. Cytosolic Ca^{2+} concentration ($[Ca^{2+}]_{cyt}$) variations were measured as in figure 1B. 30 µM 2-APB was added 30s prior Ca^{2+} ion readdition.
